# La leishmaniose viscérale chez l'adulte à propos de douze cas

**DOI:** 10.11604/pamj.2016.23.194.8921

**Published:** 2016-04-15

**Authors:** Imane Benbella, Fatima Aich, Majdouline Elkhiyat, Hanane Khalki, Assya Khermach, Imane Bergui, Imane Tlemçani, Moncef Amrani Hassani

**Affiliations:** 1 Service d'Hématologie, Laboratoire des Analyses Médicales, Centre Hospitalier Universitaire de Fès, Maroc

**Keywords:** Leishmaniose viscérale, adulte, immunocompétent, Visceral leishmaniasis, adult, immunocompetent

## Abstract

La leishmaniose viscérale est une maladie à transmission vectorielle liée essentiellement, au niveau de pourtour méditerranéen, à l'infection par leishmania infantum. Habituellement rare chez l'adulte, sa prévalence a récemment connu une augmentation y compris chez les sujets immunocompétents. Le but de notre étude est de présenter le profil épidémiologique de la leishmaniose viscérale chez l'adulte ainsi que l'importance du diagnostique biologique dans l'identification de cette maladie. Notre étude s'est étendue sur six ans de Janvier 2009 à Janvier 2014, et a colligé douze patients hospitalisés au Centre Hospitalier Universitaire Hassan II de Fès. L'altération de l’état général et la splénomégalie ont dominé le tableau clinique. Sur le plan biologique, l'anémie a été quasi constante. La confirmation diagnostique a consisté en la mise en évidence du parasite au niveau de la moelle. L’évolution sous traitement a été favorable pour tous nos patients. Ainsi, la recrudescence que connait la leishmaniose viscérale chez l'adulte et son tableau clinique peu spécifique doit la faire évoquer devant toute splénomégalie fébrile, afin de permettre un diagnostic et une prise en charge thérapeutique précoces.

## Abstract (en)

Visceral leishmaniasis is a vector-borne disease essentially associated with Leishmania infantum infection in the Mediterranean basin. Although rare in adults, its prevalence has recently increased even among immunocompetent individuals. The aim of our study is to reveal the epidemiological features of visceral leishmaniasis in adults and the importance of biological diagnostic in the identification of this disease. Our study spanned six years from January 2009 to January 2014 and data were collected from twelve patients hospitalized at University Hospital Hassan II, Fez. Alteration of general state and splenomegaly dominated the clinical picture. Biologically, anemia was almost constant. Diagnosis was confirmed by parasite identification at the level of bone marrow. The response to treatment was favorable for all our patients. Thus, visceral leishmaniasis recrudescence in adults and its nonspecific clinical picture must lead the clinicians to suspect it when fever accompanying splenomegaly occurs, thus enabling early diagnosis and therapeutic management.

## Introduction

La leishmaniose est une antropozoonose causée par des protozoaires flagellés du genre *Leishmania* qui sont transmis par la piqûre d'un insecte diptère hématophage de 2 à 4mm de long: le phlébotome femelle [1, 2]. Leishmania infantum est habituellement responsable de la leishmaniose viscérale au niveau du pourtour méditerranéen [3]. L'incidence mondiale des leishmanioses viscérales est estimée à 500.000 cas/an, elles connaissent une large distribution géographique avec l'apparition de nouveaux foyers même dans les pays initialement connus indemnes [1–4]. C'est une maladie principalement infantile. Sa survenue chez l'adulte immunocompétent est rare. Le tableau clinique se distingue par des signes moins patents que chez l'enfant [2]. Notre étude vise à discuter du profil épidémiologique de la leishmaniose viscérale chez l'adulte au Maroc, et de mettre en évidence l'importance du diagnostic biologique dans l'identification de la maladie.

## Méthodes

Il s'agit d'une étude rétrospective réalisée sur une période de six ans s’étendant de Janvier 2009 à Janvier 2014, colligée au niveau du Centre Hospitalier Universitaire Hassan II de Fès. Les données épidémiologiques, cliniques, biologiques et thérapeutiques ont été obtenues à partir des dossiers médicaux des patients chez qui une leishmaniose fut diagnostiquée. Ainsi les éléments suivant ont été recherchés: la présence d'une fièvre définie par une température centrale supérieure à 37,8^°^C, et/ou d'une hépato-splénomégalie; la présence d'une anémie (hémoglobine < 12 g/dl) associée ou non à une thrombopénie (plaquettes < 150.000/mm^3^) et/ou à une leucopénie (leucocytes < 4000/mm^3^); la mise en évidence des leishmanies (formes amastigotes) par examen direct d'un frottis de moelle osseuse colorée au May-Grünwald-Giemsa. Par ailleurs, tous les patients ont bénéficié d'un hémogramme, un bilan rénal, un bilan hépatique, un bilan inflammatoire et une sérologie VIH. Une seule molécule a été utilisée pour le traitement. Il s'agit de l'antimoniate de meglumine. Une surveillance clinico-biologique a été pratiquée. Les critères de guérison après traitement ont été la disparition des signes cliniques et la correction des anomalies biologiques.

## Résultats

**Figure 1:**
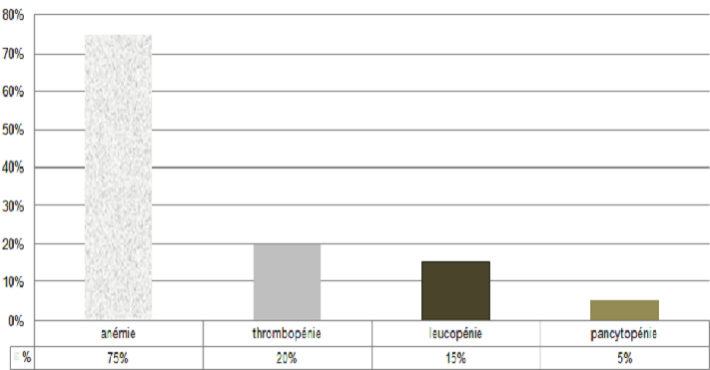
Répartition des différentes anomalies biologiques retrouvées chez les patients adultes atteint de leishmaniose viscérale.

**Figure 2:**
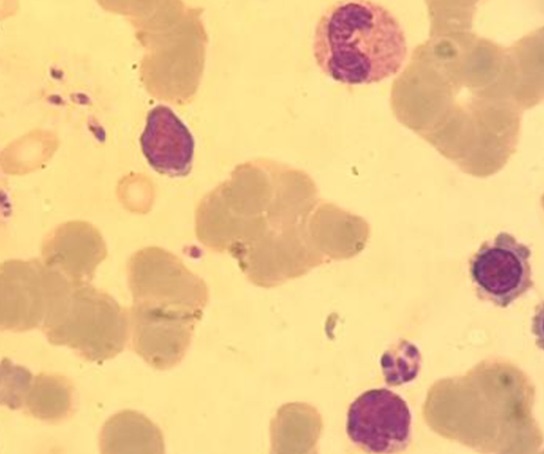
Présence de corps de leishmanies sous forme amastigote en extra cellulaire au myélogramme (×100)

**Figure 3:**
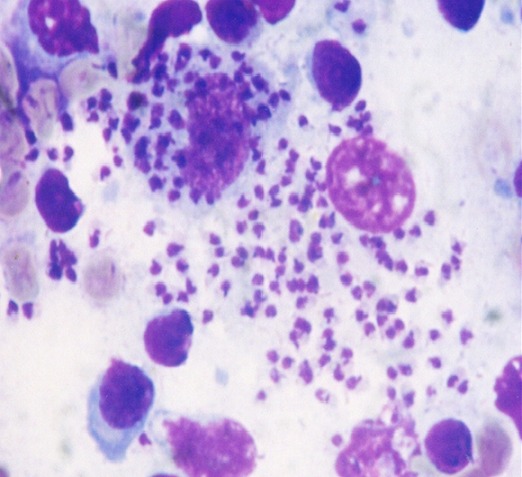
Présence au niveau du myélogramme de corps de leishmanies sous forme amastigote en extra cellulaire et en intra cellulaire (×100)

Au cours de la période de notre étude, douze patients hospitalisés au Centre Hospitalier Universitaire Hassan II de Fès ont été recensés, dont huit hommes et quatre femmes. Leur moyenne d’âge se situait à 42 ans (17 à 50 ans). Tous les patients étaient originaires du centre et du sud du pays. Les patients ne présentaient pas d'antécédents pathologiques notables. La sérologie pour le VIH a été négative dans tous les cas, et aucune autre cause d'immunodépression n'a été relevée. En moyenne le délai entre le début de la symptomatologie et l'hospitalisation étaient de 25 jours (14 jours-36 jours). Le tableau clinique a été dominé par la fièvre (100%), la splénomégalie (58%), et l'altération de l’état général avec un amaigrissement et une asthénie (58%) (Tableau 1). Sur le plan biologique, l'anémie a été constante, une leucopénie a été retrouvée dans 15% des cas et une thrombopénie a été notée dans 20% des cas (Figure 1). Le diagnostic a été confirmé par la mise en évidence du parasite dans la moelle (Figure 2, Figure 3). Tous nos patients avaient une moelle envahie par les corps de leishmanies. En effet, le frottis de la moelle osseuse coloré au May Grünwald Giemsa a permis de mettre en évidence des corps de leishmanies sous formes amastigotes, en extra cellulaire dans dix cas, et en intra cellulaire dans deux cas. Tous les patients ont bénéficié d'un traitement à base d'antimoniate de méglumine pour une durée de 14 à 28 jours. Ils ont tous bénéficié d'une seconde cure de consolidation six semaines plus tard, pour une durée de 15 jours, avec une évolution favorable chez tous les patients.

**Tableau 1:** Répartition des différents signes cliniques retrouvés chez les patients adultes atteint de leishmaniose viscérale

Signes cliniques	Effectif	Pourcentage (n = 12)
**fièvre**	12	100%
**splénomégalie**	7	58%
**amaigrissement**	7	58%
**pâleur**	4	33%
**arthralgies**	4	33%
**adénopathies**	1	8%
**hépatomégalie**	3	25%
**Asthénie + anorexie**	3	25%

## Discussion

Les leishmanioses sont des maladies à transmission vectorielle liées à l'infection par un protozoaire flagellé appartenant au genre *Leishmania* [4] qui sont transmis par la piqûre d'un insecte diptère vecteur hématophage: le phlébotome femelle [5]. Selon l'espèce parasitaire en cause et les mécanismes de défense mis en place par l'hôte, la maladie peut prendre la forme d'une affection tégumentaire ou systémique [4]. On distingue trois entités cliniques: cutanéo-muqueuse, cutanée et la leishmaniose viscérale [1]. La leishmaniose viscérale est présente dans 69 pays répartis sur tous les continents à l'exception de l'Océanie [4, 6]. Elle est habituellement causée par *L. donovani* dans le sous continent indien et en Afrique de l'Est, et par *L. infantum* sur le pourtour méditerannéen, en Asie centrale, en Chine et en Amérique du Sud. Beaucoup plus rarement d'autres espèces peuvent donner des atteintes viscérales comme *L. tropica* au Moyen Orient ou en Amérique latine [4]. L'incidence mondiale des leishmanioses viscérales est estimée à 500.000 cas/an, entrainant 50.000 décès annuels [4, 6]. Dans les trois pays du Maghreb (Maroc, Algérie, Tunisie), elle se développe dans 95% des cas chez des enfants de moins de cinq ans [1]. Rare chez l'adulte, sa prévalence est en nette progression. Cette modification du profil épidémiologique de la leishmaniose viscérale méditerranéenne peut s'expliquer par l'association de plusieurs facteurs dominés par la multiplication des causes d'immunodépression, qu'elles soient virales comme l'infection par le VIH, ou iatrogènes induites par certains médicaments comme les corticoïdes, les immunosuppresseurs et les anti-mitotiques [2, 7]. Dans les pays sud européens, l'atteinte des adultes est fréquemment liée à l'infection par le VIH. En effet, dans ces pays, on estime que près de 9% des personnes infectées par le VIH développent une leishmaniose viscérale. Par ailleurs, la diminution du contact homme-parasite, en rapport principalement avec l'urbanisation, a fait que les populations sont devenues moins immunisées et par conséquent davantage susceptibles à l'infection au-delà de l'enfance [2].

Dans notre étude, aucun des patients n’était infecté par le VIH, et aucune autre cause d'immunodépression n'a été retrouvée. Toutefois, nous n’éliminons pas la possibilité de la présence d'une discrète immunodéficience qui n'a pu être relevée par nos investigations. Notre étude a également relevé une prédominance masculine, ce qui rejoint les données de la littérature. Ces résultats peuvent être expliqués par le fait que l'homme porte souvent des habits peu couvrants et s'expose d'avantage à la piqûre du phlébotome [2]. Chez l'adulte, même immunocompétent, la présentation clinique est moins évocatrice, que chez l'enfant: la fréquence de la splénomégalie et de la fièvre dépassent à peine 80% des cas selon les séries, en particulier en cas d'infection à *L. infantum* [2, 4]. Dans notre étude, tous les patients présentaient une fièvre et 58% une splénomégalie (Tableau 1). A ces anomalies cliniques, viennent s'ajouter des perturbations biologiques. Ainsi, une cytopénie est le plus souvent retrouvée et peut toucher les trois lignées [2, 4, 8]. Dans notre étude, l'atteinte de la lignée érythrocytaire a prédominé, avec une anémie quasi constante (75%). L’électrophorèse des protéines sériques ainsi que la mesure de la vitesse de sédimentation ont également contribué à l'orientation diagnostique. En effet, un syndrome inflammatoire est souvent retrouvé. Ce dernier regroupe fréquemment: une hyperprotidémie, une hypergammaglobulinémie polyclonale avec un rapport albumine/globuline souvent inversé et une vitesse de sédimentation accélérée [1, 2]. Une très forte présomption diagnostique repose sur la positivité de la sérologie, plusieurs techniques ont été développées notamment l'hémagglutination directe et les bandelettes rK39 (immunochromatographie). Ces deux méthodes d'un excellent rendement ont respectivement une sensibilité de 93.9% et de 35% et une spécificité de 85.9% [2, 4, 9]. Classiquement, le diagnostic nécessite un prélèvement de moelle osseuse, avec réalisation d'un frottis coloré au May Grünwald Giemsa. L'examen direct met en évidence les formes amastigotes typiquement au sein de cellules phagocytaires. Le parasite de petite taille (2-5µm) est caractérisé par la présence simultanée du noyau rond ou ovalaire et du kinétoplaste punctiforme ou de forme allongée [4]. Quand il est disponible, le diagnostic moléculaire est un excellent outil du diagnostic, du suivi post thérapeutique des leishmanioses et d’étude des sujets asymptomatiques porteurs du parasite. La PCR détecte des parasitémies < 1 parasite/ml. La sensibilité est similaire sur les prélèvements sanguins. La PCR permet également d'identifier l'espèce voire la souche parasitaire [4, 10].

Les dérivés de l'antimoine demeurent le traitement de référence de la leishmaniose viscérale dans de nombreux pays [2]. La posologie d'antimoine recommandée par l'OMS est de 20mg/kg/j. La voie intramusculaire est recommandée à cause de sa plus faible toxicité. La durée du traitement recommandée est de 28 jours. Des effets indésirables sont observés dans environ 15% des cas [2]. Des échecs de traitement récemment rapportés notamment en Inde, en France et en Italie laissent supposer l'existence d'une résistance des leishmanies vis-à-vis de l'antimoine [4]. Ainsi de nouvelles perspectives thérapeutiques ont été proposées. Le traitement par l'amphotéricine B qui est un inhibiteur de la synthèse des stérols constituants la membrane des leishmanies, représente une alternative aux dérivés stibiés [2]. En effet, l'amphotéricine B a montré une excellente efficacité avec des taux de succès dépassant 95% chez l'immunocompétent pour des posologies totales de 10 à 20mg/kg [4, 11]. Néanmoins, elle présente de nombreux effets indésirables limitant sont utilisation, notamment ceux liés à la perfusion, la toxicité cardiaque, l'hypocalcémie et l'insuffisance rénale [12]. L'utilisation de l'amphotéricine B, associée à des complexes lipidiques, a permis d'augmenter les doses thérapeutiques, de réduire la durée du traitement et les effets secondaires, et d'obtenir par conséquent une meilleure efficacité [2, 13].

Plus récemment, de nouvelles méthodes efficaces par voie orale, la sitamaquine et surtout la miltéfosine ont été utilisées avec succès dans le traitement de la leishmaniose viscérale [2, 14]. Dans notre étude, l’évolution de nos patients traités par dérivés d'antimoine a été marquée par la survenue de la guérison en absence de tout effet indésirable ou de signes d'intolérance.

## Conclusion

La leishmaniose viscérale est en recrudescence chez l'adulte au Maroc. Elle doit être évoquée devant toute splénomégalie fébrile en zone endémique. Les données biologiques sont peu spécifiques. Le diagnostic de certitude repose sur la mise en évidence du parasite dans les liquides biologiques. L'instauration rapide d'un traitement spécifique est vitale. Toutefois, le meilleur moyen de lutte contre cette zoonose reste préventif par le dépistage et le traitement précoce des malades et l’éradication des vecteurs et du réservoir des parasites (chiens).

### Etat des connaissances sur le sujet

- La leishmaniose viscérale est une antropozoonose causée par des protozoaires flagellés du genre leishmania
- Leishmania infantum est le plus souvent responsable des leishmanioses viscérales au niveau du pourtour méditérranéen
- La leishmaniose viscérale touche essentiellement l'enfant

### Contribution de notre étude a la connaissance

- La leishmaniose viscérale est rare chez l'adulte, néanmoins ces dernières années son incidence a connu une recrudescence du nombre de cas recensés
- La leishmaniose viscérale chez l'adulte peut survenir même en l'absence de toute cause d'immunodépression
- La symptomatologie chez l'adulte est le plus souvent atypique, néanmoins il faut penser à évoquer ce diagnostic
